# Supplementary material for: Galangin and 1′-Acetoxychavicol Acetate from Galangal (Alpinia galanga) Suppress Lymphoma Growth via c-Myc Downregulation and Apoptosis Induction
Source: Biology (Basel). 2025 Aug 21;14(8):1098. doi: 10.3390/biology14081098 (PMC12384025; doi:10.3390/biology14081098)
Supplement: Supplementary file 1 [file biology-14-01098-s001.zip › biology-3793103 Supplementary Materials/Revised Supplementary Figures.pdf]

Supplementary Figures

(a)

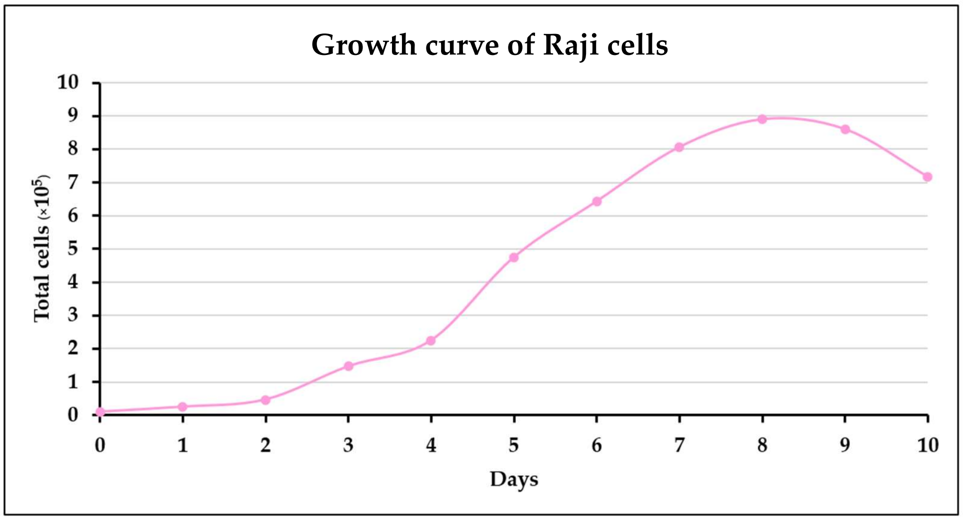

(b)

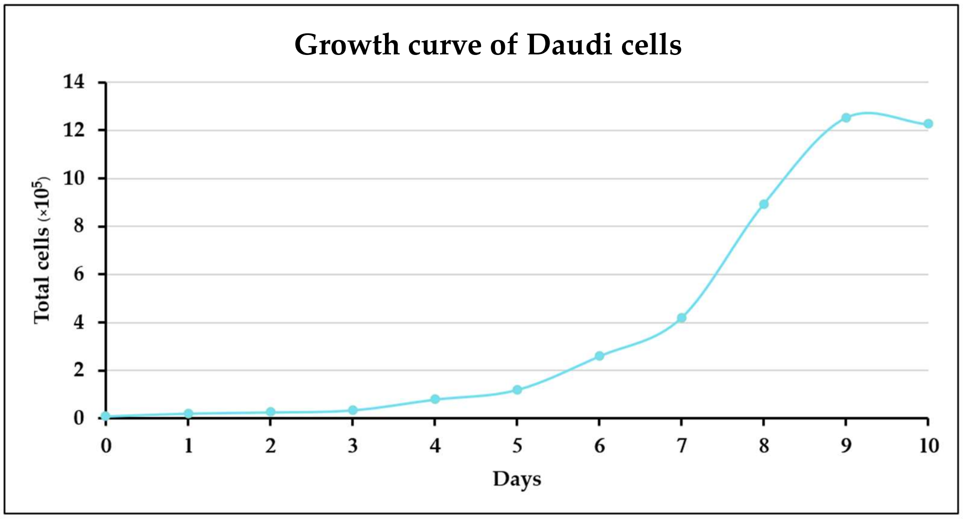

Supplementary Figure S1. Cell growth kinetics of Raji and Daudi cells over a 10-day period, assessed by the trypan blue exclusion assay. (**a**) Growth curve of Raji cells. (**b**) Growth curve of Daudi cells. Cells were cultured under standard conditions and counted daily using trypan blue staining to distinguish viable cells. Viable cell numbers were plotted over time to evaluate proliferation dynamics.

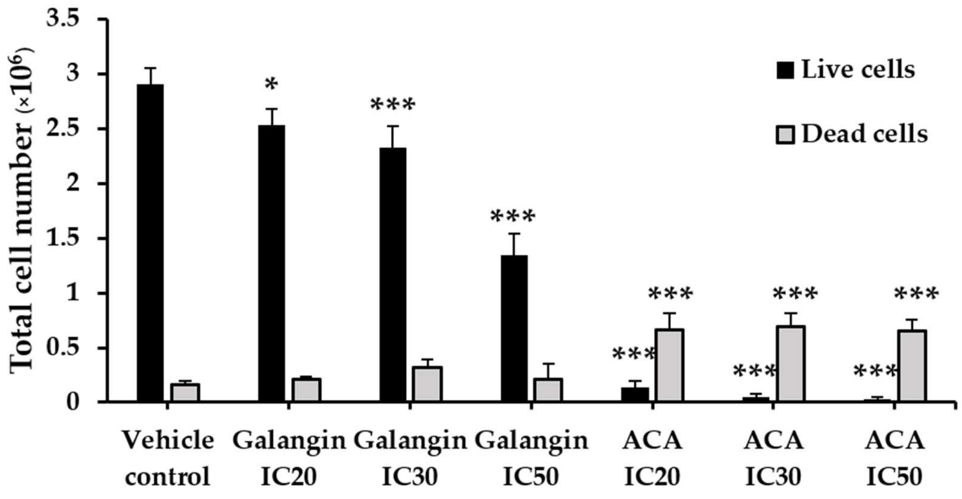

Supplementary Figure S2. Total cell numbers of Daudi cells after 48 h of treatment with galangin and ACA at IC_{20}, IC_{30}, and IC_{50}, determined by the trypan blue exclusion method. Asterisks (*) indicate significant differences compared with vehicle control (*p<0.05, **p<0.01, ***p<0.001).

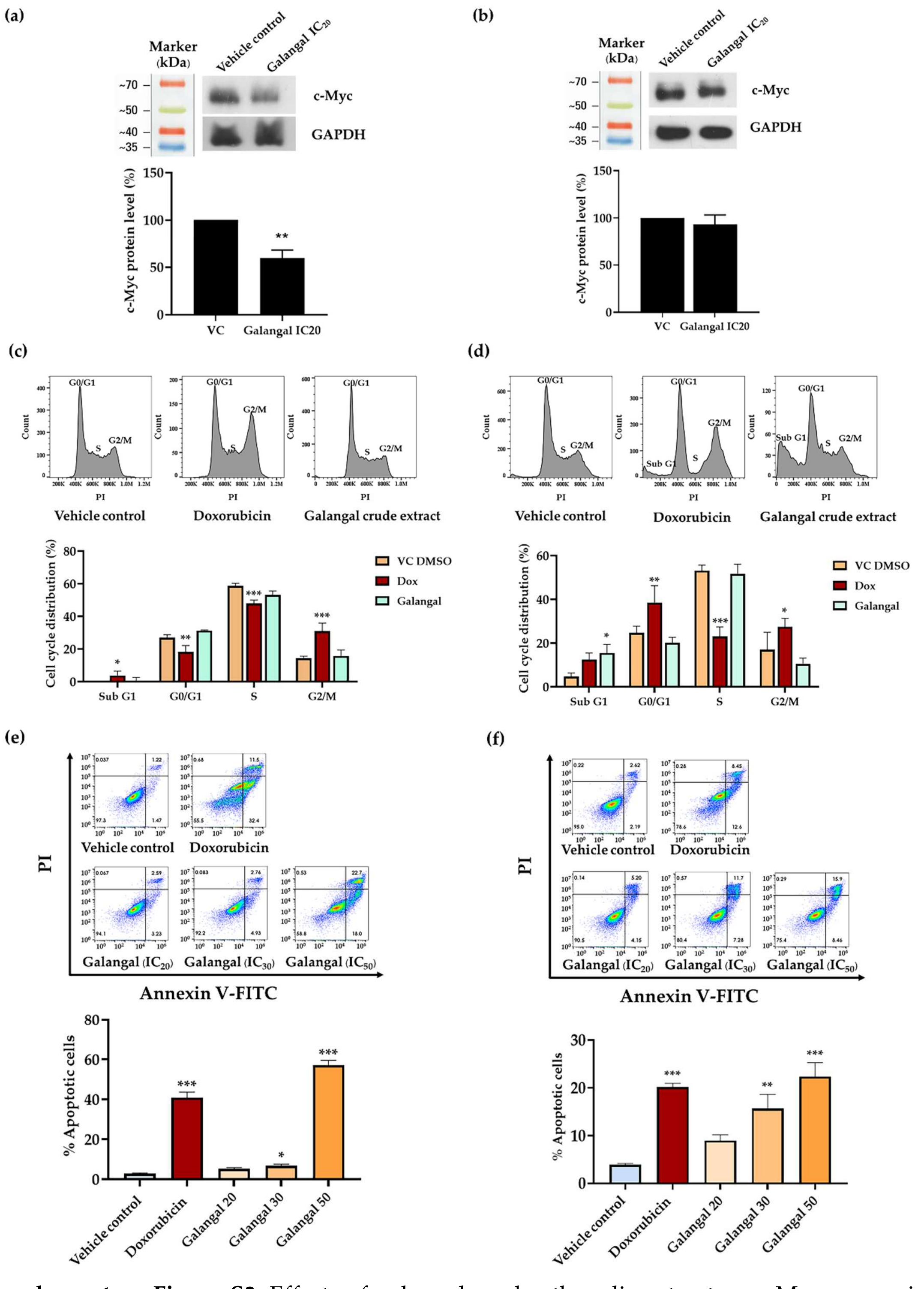

Supplementary Figure S3. Effects of galangal crude ethanolic extract on c-Myc expression, cell cycle progression, and apoptosis in Raji and Daudi cells. (**a**, **b**) c-Myc protein levels in Raji and Daudi cells, respectively, after treatment with galangal crude ethanolic extract at the IC_{20} concentration for 48 h. (**c**, **d**) Cell cycle distribution of Raji and Daudi cells, respectively, after treatment at the IC_{20} concentration for 48 h, analyzed by flow cytometry. (**e**, **f**) Apoptosis analysis of Raji and Daudi cells, respectively, after treatment at the IC_{20}, IC_{30}, and IC_{50}

concentrations for 48 h, assessed by Annexin V/PI staining. Data represent the mean ± SD from three independent experiments. Statistical significance was determined using appropriate tests ($p < 0.05$).

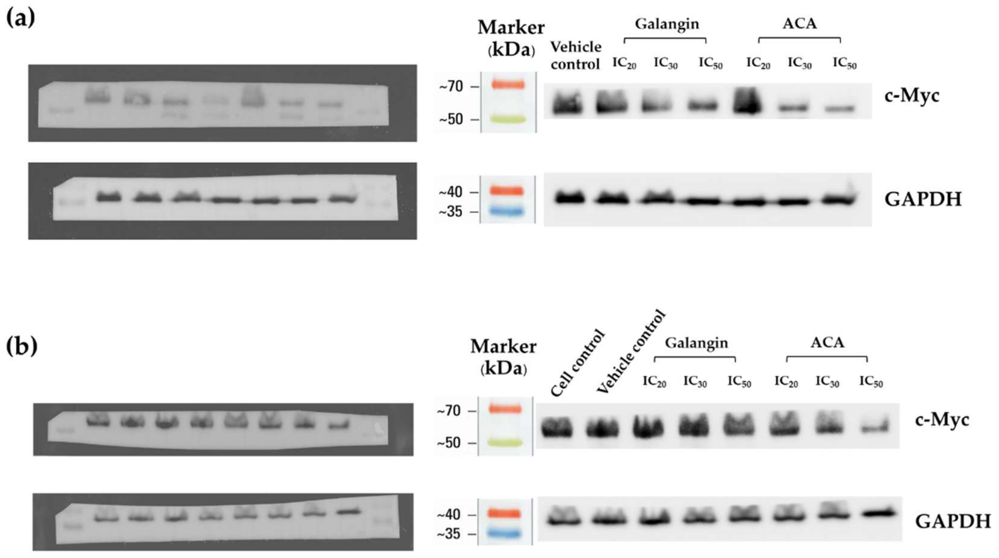

Supplementary Figure S4. Uncropped Western blot membranes corresponding to c-Myc and GAPDH. (**a**) c-Myc protein levels in Raji cells after treatment with galangin and ACA at IC_{20}, IC_{30}, and IC_{50} concentrations for 48 h. (**b**) c-Myc protein levels in Daudi cells after treatment with galangin and ACA at IC_{20}, IC_{30}, and IC_{50} concentrations for 24 h.

(a)

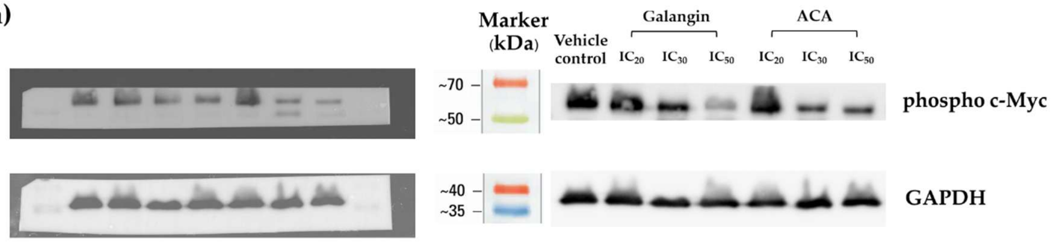

(b)

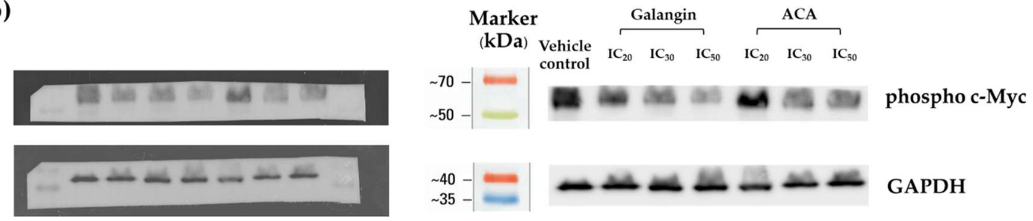

Supplementary Figure S5. Uncropped Western blot membranes corresponding to p-c-Myc and GAPDH. (**a**) p-c-Myc protein levels in Raji cells after treatment with galangin and ACA at IC_{20}, IC_{30}, and IC_{50} concentrations for 48 h. (**b**) p-c-Myc protein levels in Daudi cells after treatment with galangin and ACA at IC_{20}, IC_{30}, and IC_{50} concentrations for 24 h.

(a)

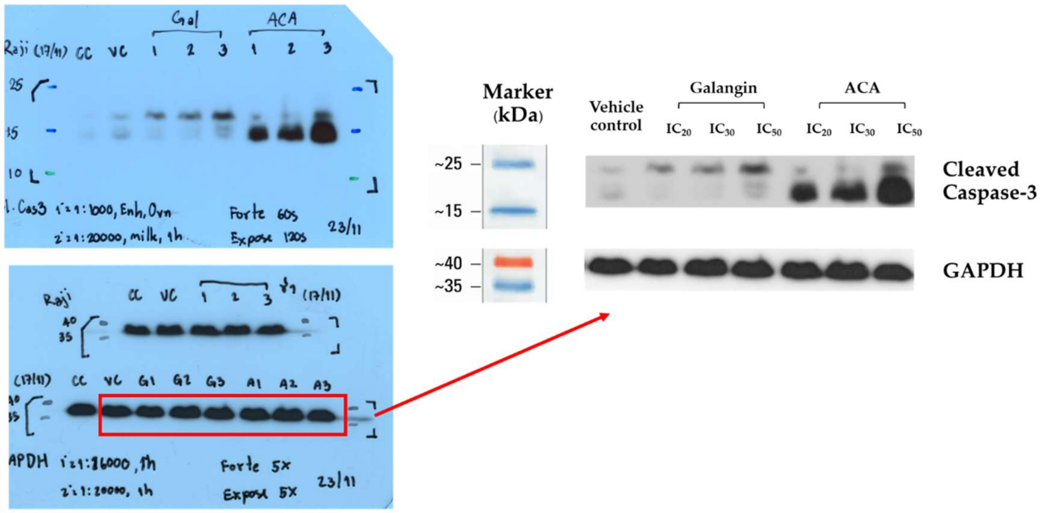

(b)

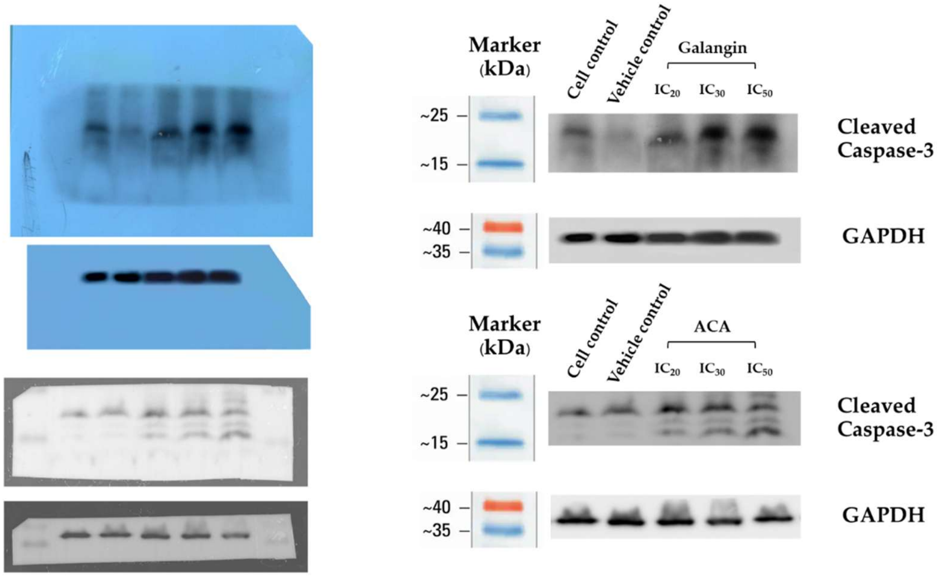

Supplementary Figure S6. Uncropped Western blot membranes corresponding to Cleaved caspase-3 (cl-casp3) and GAPDH. **(a)** Cl-casp3 protein levels in Raji cells (1.0 × 10^5 cells/ml) after treatment with galangin and ACA at IC_{20}, IC_{30}, and IC_{50} concentrations for 48 h. **(b)** Cl-Casp3 protein levels in Daudi cells (2.0 × 10^5 cells/ml) after treatment with galangin and ACA at IC_{20}, IC_{30}, and IC_{50} concentrations for 24 h.

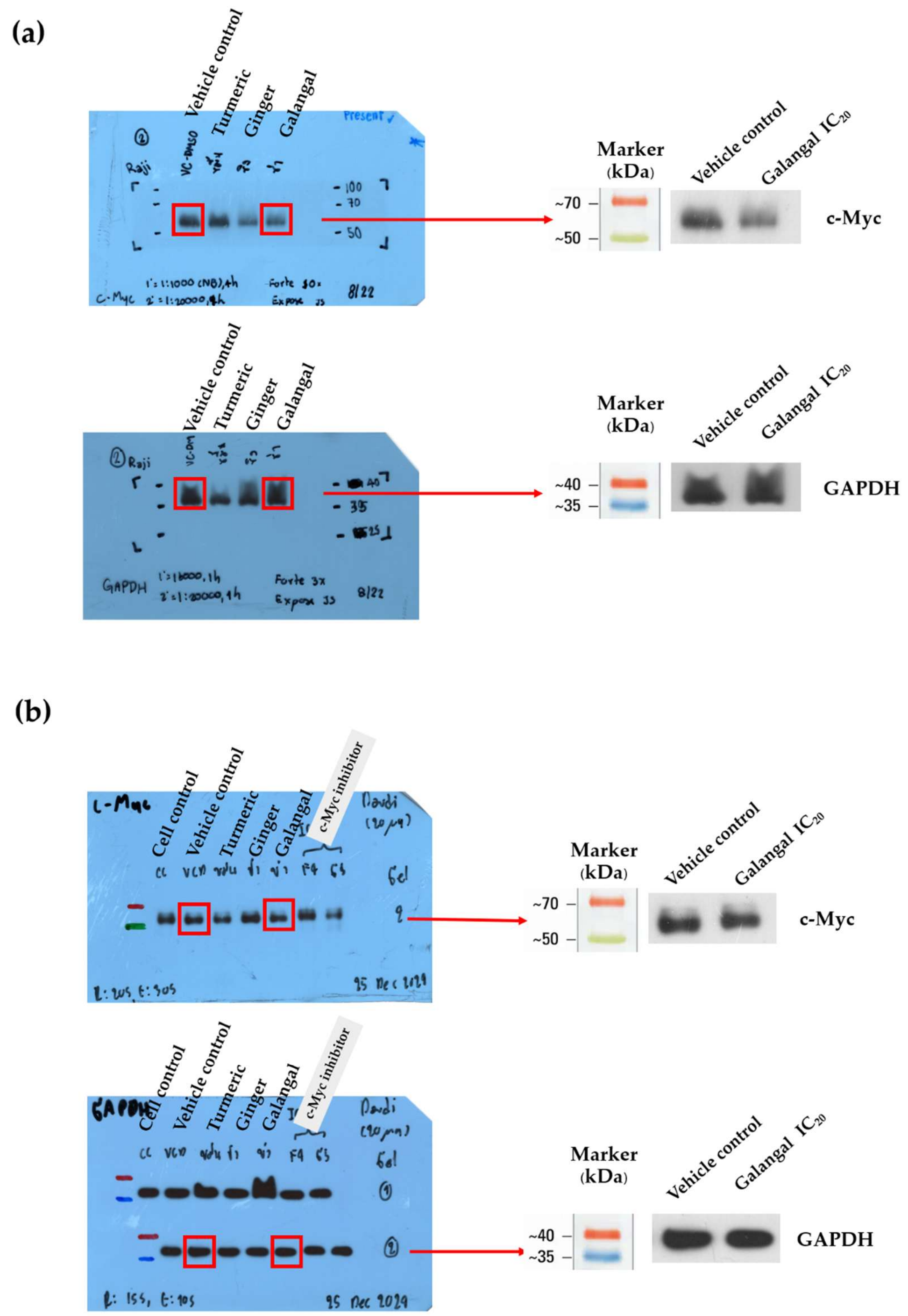

Supplementary Figure S7. Uncropped Western blot membranes corresponding to c-Myc and GAPDH. (**a**) c-Myc protein level in Raji cells after treatment with galangal crude extract at IC_{20} concentration for 48 h. (**b**) c-Myc protein level in Daudi cells after treatment with galangal crude extract at the IC_{20} concentration for 48 h.
