# Supplementary figures and images for: Galangin and 1′-Acetoxychavicol Acetate from Galangal (Alpinia galanga) Suppress Lymphoma Growth via c-Myc Downregulation and Apoptosis Induction
Source: Biology (Basel). 2025 Aug 21;14(8):1098. doi: 10.3390/biology14081098 (PMC12384025; doi:10.3390/biology14081098)

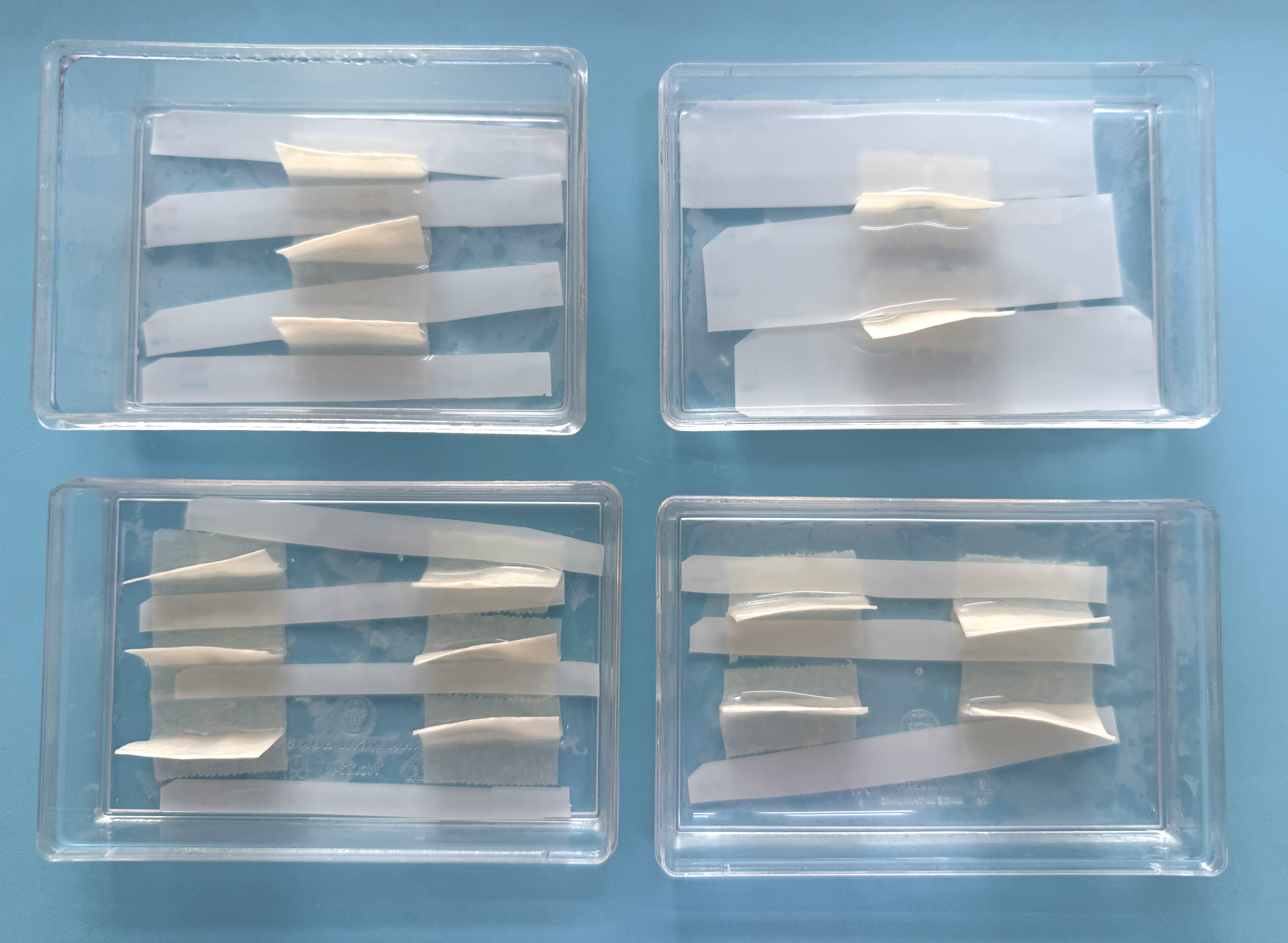

Supplement: Supplementary file 1 [file biology-14-01098-s001.zip › biology-3793103 Supplementary Materials/Cutting membranes.JPEG]

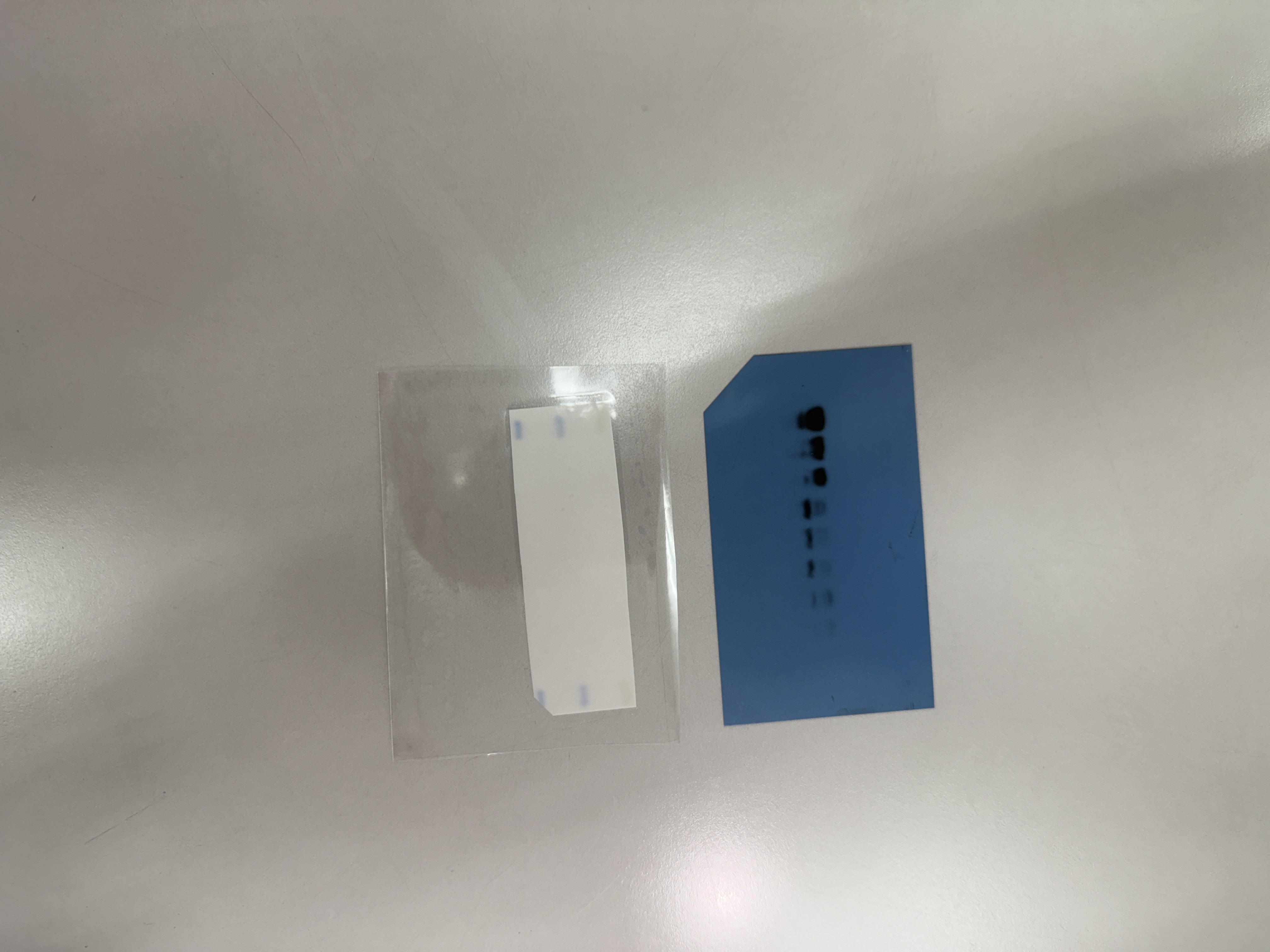

Supplement: Supplementary file 1 [file biology-14-01098-s001.zip › biology-3793103 Supplementary Materials/Figure 5.JPEG]

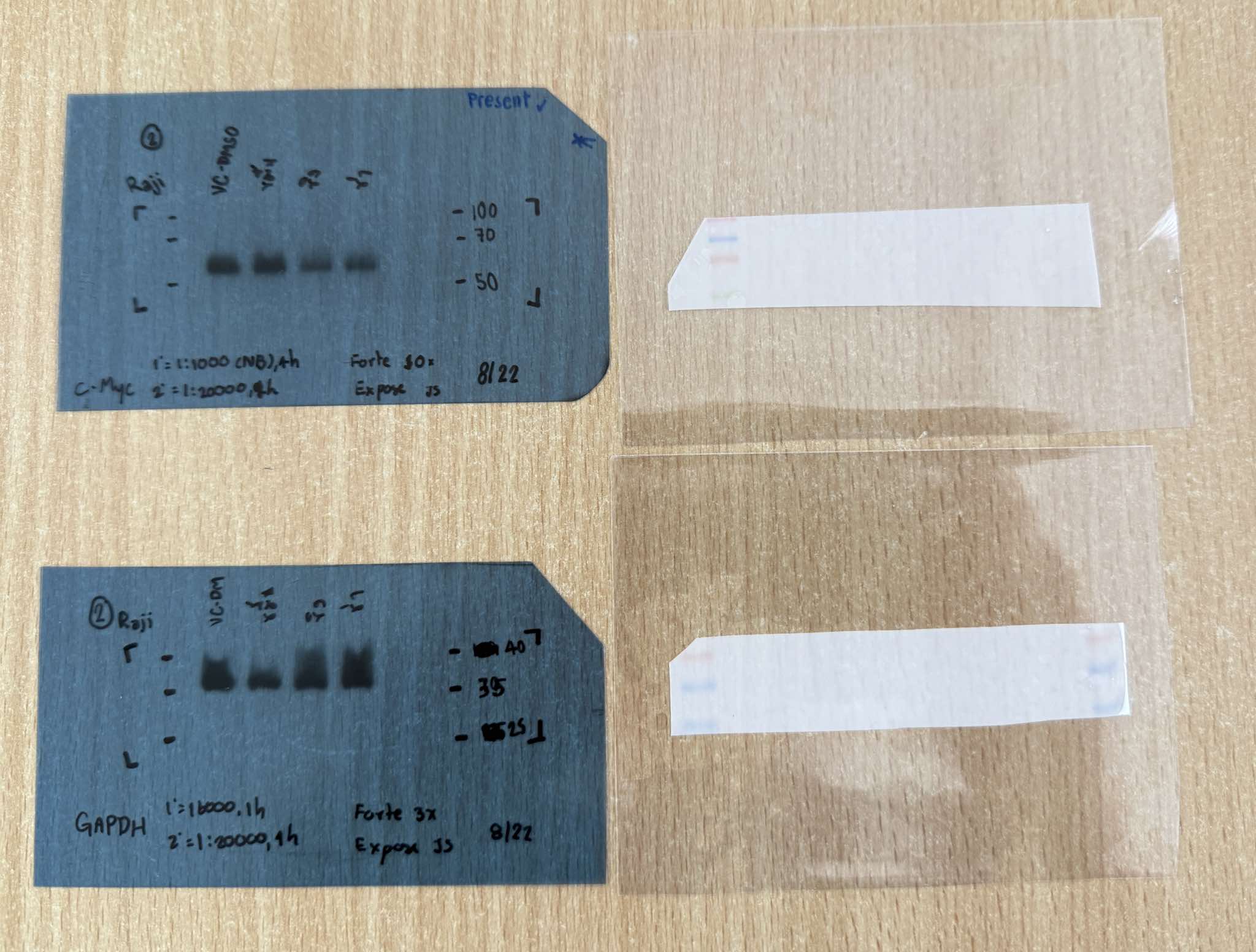

Supplement: Supplementary file 1 [file biology-14-01098-s001.zip › biology-3793103 Supplementary Materials/Supplementary Figure S7a.jpg]

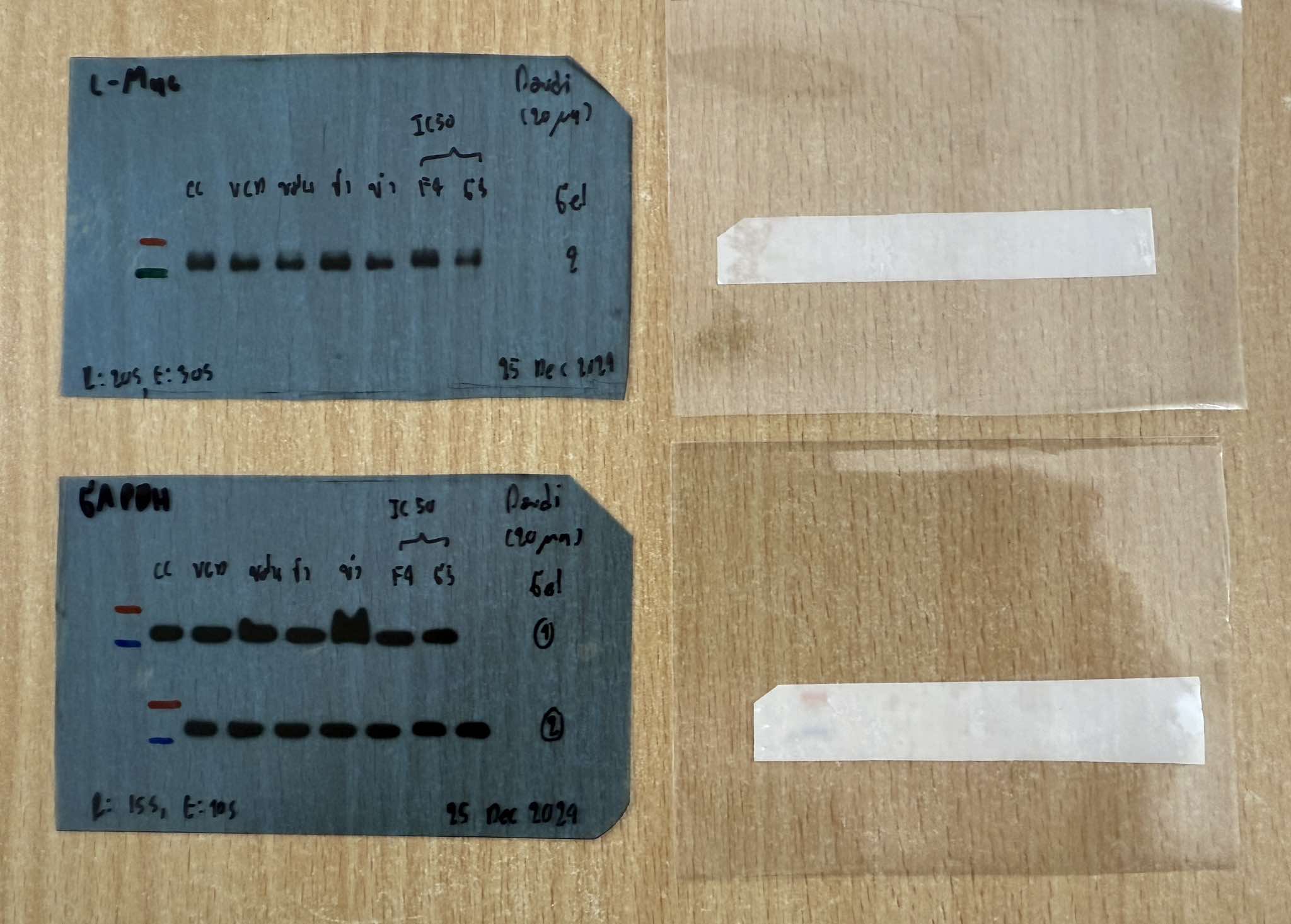

Supplement: Supplementary file 1 [file biology-14-01098-s001.zip › biology-3793103 Supplementary Materials/Supplementary Figure S7b.jpg]
